# Evaluating the therapeutic impact of tirzepatide in people with partial lipodystrophy

**DOI:** 10.1210/clinem/dgag126

**Published:** 2026-03-20

**Authors:** Mandour O Mandour, Anna Stears, Agatha Van Der Klaauw, Catherine Flanagan, Lisa Gaff, Charlotte Jenkins, David B Savage

**Affiliations:** University of Cambridge Metabolic Research Laboratories, Wellcome Trust-MRC Institute of Metabolic Science, Cambridge CB2 0QQ, UK; National Severe Insulin Resistance Service, Wolfson Diabetes and Endocrinology Clinic, Cambridge University Hospitals NHS Trust, Cambridge CB2 0QQ, UK; University of Cambridge Metabolic Research Laboratories, Wellcome Trust-MRC Institute of Metabolic Science, Cambridge CB2 0QQ, UK; National Severe Insulin Resistance Service, Wolfson Diabetes and Endocrinology Clinic, Cambridge University Hospitals NHS Trust, Cambridge CB2 0QQ, UK; National Severe Insulin Resistance Service, Wolfson Diabetes and Endocrinology Clinic, Cambridge University Hospitals NHS Trust, Cambridge CB2 0QQ, UK; National Severe Insulin Resistance Service, Wolfson Diabetes and Endocrinology Clinic, Cambridge University Hospitals NHS Trust, Cambridge CB2 0QQ, UK; University of Cambridge Metabolic Research Laboratories, Wellcome Trust-MRC Institute of Metabolic Science, Cambridge CB2 0QQ, UK

**Keywords:** partial lipodystrophy, tirzepatide, insulin resistance, diabetes, dual GIP/GLP-1 receptor agonist

## Abstract

**Background:**

Lipodystrophy syndromes (LD) are a group of rare conditions characterized by the generalized or partial absence and/or dysfunction of adipocytes. Due to the lack of fat storage capacity, excess lipid accumulates in other tissues, leading to severe insulin resistance. At present, effective therapeutic options remain limited, with metreleptin currently the only specific licensed treatment. To this end, we evaluated the therapeutic impact of tirzepatide in people with partial lipodystrophy (PLD).

**Methods:**

This was a single-center, retrospective, observational study, including all patients with PLD treated with tirzepatide between January 2022 and September 2025.

**Results:**

Forty patients with PLD were included FPLD1 like = 26 (65%), FPLD2 = 4 (10%), FPLD3 = 7 (17.5%), FPLD7 = 1 (2.5%), APLD = 1 (2.5%), unclassified = 1 (2.5%). 87.5% (*n* = 35) were female with a median age of 47 years (IQR 39—59) and a median BMI of 31.5 kg/m^2^ (IQR 27.3 to 35.5). After a median 9.5 month follow-up (IQR 7 to 11), marked reductions were seen in body weight from 91.0 kg (IQR 75.0 to 106.0) to 82.8 kg (IQR 69.0 to 93.4; *P* < .0001), in HbA1c from 65 mmol/mol (IQR 54.5 to 85) to 52 mmol/mol (IQR 39 to 62; *P* < .0001) and in serum triglycerides from 2.80 mmol/L (IQR 1.6 to 3.7) to 1.83 mmol/L (IQR 1.0 to 2.9; *P* = .0005). Daily insulin requirements fell from 177.5 IU (IQR 101.3 to 213.8) to 58.0 IU (IQR 0 to 112.5; *P* = .0028).

**Conclusion:**

In patients with partial lipodystrophy, tirzepatide use resulted in substantial statistically significant reductions in weight, glycemic control, serum triglycerides, and daily insulin requirement. We recommend further prospective studies to support these findings and to evaluate its impact earlier in the course of the disorder.

Lipodystrophy syndromes (LD) are a group of conditions characterized by the absence and/or dysfunction of white adipose tissue. Classification is typically based on the origin (ie, inherited or acquired) and pattern of fat loss (ie, localized, partial, or generalized). In general, monogenic inherited forms of LD are all rare, but recent findings have indicated that familial partial lipodystrophy 1 (FPLD1) is likely to be polygenic in most cases and is, in fact, akin to an extreme form of widely prevalent “apple-shape” or central adiposity (1). It is also increasingly apparent that subtle forms of partial lipodystrophy (PLD) are a common cause of nonketotic diabetes (2, 3).

The reduction in fat mass in lipodystrophy results in a severe or relative decrease in circulating leptin levels, leading to hyperphagia (4). As surplus energy cannot be stored optimally in adipose tissue in this setting, fat accumulates in other organs less well adapted to store surplus lipid, such as the liver, skeletal muscle, and pancreas. This “ectopic fat” induces a toxic response (known as lipotoxicity) which has been implicated in causing insulin resistance, exacerbating steatotic liver disease, and impairing pancreatic beta cell function (5-7).

Diagnosis is multifaceted, combining clinical detection of altered adipose tissue mass and distribution, radiological imaging, laboratory testing, and molecular genetics. Treatment focuses on managing metabolic disease and complications using similar approaches to those used to treat metabolic disease linked to obesity. Dietary calorie restriction is particularly important and effective, but challenging to implement given the increased drive to eat due to low or relatively low leptin concentrations. Metreleptin remains the only licensed drug approved for the treatment of lipodystrophy to date and is very effective in alleviating the burden of metabolic disease in patients with generalized lipodystrophy (GLD) (8). However, metreleptin is expensive, and its impact in patients with partial lipodystrophy (PLD) is variable and considerably less marked than that in patients with GLD (9).

Leptin's efficacy largely relates to its impact on reducing calorie intake (10). In keeping with this notion, bariatric surgery has been shown to be very effective in ameliorating metabolic disease risk in patients with PLD (11-13). GLP1 agonists such as liraglutide, dulaglutide, and semaglutide have also been reported to be associated with weight loss and improved metabolic control in people with partial lipodystrophy (14-18). Tirzepatide is a dual GIP/GLP1 RA (glucagon-like peptide/glucose-dependent insulinotropic polypeptide receptor agonist), which appears to be more effective than other GLP1 receptor agonists in reducing energy intake, body weight, and its associated metabolic disease states in people with obesity and type 2 diabetes (19-21). Thus, we considered tirzepatide to be a rational therapeutic option in patients with PLD.

## Methods

This was a single-center, retrospective, observational study including all patients with partial lipodystrophy (PLD) seen in the Severe Insulin Resistance Service at Cambridge University Hospital Foundation Trust between January 2022 and September 2025 who were treated with tirzepatide. Access to patient clinical records for this retrospective analysis was approved by CUHFT R&D (Clinical project ID 6069 PRN12069). Data were collected from electronic patient records.

All cases were referred to the insulin resistance/lipodystrophy specialist center in Cambridge because they were suspected to have lipodystrophy by the referring doctor. All patients were then reviewed by a specialist physician with experience in lipodystrophy in the clinic, and genetic testing was undertaken in all patients who consented to it. Genetic testing included screening for mutations in all of the following genes: AGPAT2 (NM_006412.4); BSCL2 (NM_032667.6); CAV1 (NM_001753.5); CAVIN1 (NM_012232.6); FBN1 (NM_000138.5); INSR (NM_000208.4); KCNJ6 (NM_002240.5); LIPE(NM_005357.4); LMNA (NM_170707.4); MTX2 (NM_006554.5); OTULIN (NM_138348.6); PLIN1 (NM_002666.5); POLD1 (NM_002691.4); PPARG (NM_015869.5); ZMPSTE24 (NM_005857.5). Patients with FPLD2, 3, or 7, all had genetically confirmed pathogenic mutations in LMNA, PPARG, or CAV1, respectively. Those without FPLD2, 3, 7, or acquired PLD, met the following clinical criteria which essentially correspond to those widely associated with the term FPLD1, also often known as Kobberling type PLD (22): patients with selective paucity of upper and lower limb (particularly femorogluteal) adipose tissue, preserved or increased central adiposity, insulin resistance and metabolic disease (diabetes, dyslipidemia and nonalcoholic fatty liver).

Exclusion criteria for this analysis included age <18 years and diagnosis of GLD. Baseline value was defined as results within 12 months of starting tirzepatide, and the latest value represented the most recently available values following treatment with tirzepatide. Two patients in the cohort had previously undergone bariatric surgery and were also included in the study, as they were weight stable prior to commencing tirzepatide. A further 3 patients had previously participated in a clinical study entailing a low-energy diet (800 to 1000 kcal/day). Baseline values for these individuals were the most recent values before starting tirzepatide and after completing the low-energy diet calorie restriction.

Tirzepatide was initiated at 2.5 mg weekly subcutaneous injections either by the family medicine doctor or privately obtained by some patients as it is not yet specifically licensed for use in patients with lipodystrophy in the United Kingdom. As a result, dose escalation was not standardized, and several patients remained on lower doses throughout the study period. The maximum treatment dose in the cohort was 15 mg. Baseline parameters recorded included PLD subtype, previous history of nonketotic diabetes, obstructive sleep apnea (OSA), metabolic dysfunction-associated steatotic liver disease (MASLD), hypertriglyceridemia, hypertension, polycystic ovary syndrome (PCOS), pancreatitis, cardiac disease, renal disease, peripheral vascular disease (PVD), stroke, and cirrhosis. A more detailed evaluation of retinopathy was undertaken using data from the UK National Health Service Diabetic Eye Screening Program (NHS DESP) (23). For each participant in the cohort, we sought to obtain the most recent retinal screening result prior to treatment with tirzepatide and the latest screening result following initiation of treatment. We collected retinal grading as per the NHS DESP classification, baseline HbA1c and post-tirzepatide HbA1c, which were defined as those measured within 6 months of the eye screening episode (pre- and post-tirzepatide). Worsening of retinopathy was defined as either new maculopathy or new proliferative retinopathy. Given the small sample size, analyses were descriptive and no formal statistical comparisons were performed with regard to the evolution of retinopathy. The presence of hypertriglyceridemia was defined as either a confirmed history in the patient records, current use of a triglyceride-lowering agent, or a fasting serum triglyceride level >1.70 mmol/L. Presence of hypertension was defined as either current use of antihypertensive agents or a confirmed history in the patient's electronic records. Presence of cardiac disease was defined as a history of either cardiac arrhythmia, conduction abnormalities, valve disease, coronary artery disease, or previous cardiac surgery. Presence of renal disease was defined as a documented history of chronic kidney disease, presence of proteinuria, or glomerulonephritis. Diabetes was defined by a HbA1c >48 mmol/mol (>6.5%) or presence of glycemia-lowering treatment (oral or insulin). For those on metformin monotherapy, we assessed whether this was for diabetes or to improve insulin sensitivity (or both) and the duration of diabetes was also recorded. Antidiabetic treatments assessed at baseline included, biguanides, sulfonylureas, thiazolidinediones, DPP-4 inhibitors, and SGLT2 inhibitors. In those who had previously been treated with another GLP1-RA, the median time from the previous GLP1-RA to tirzepatide was 10 months [IQR 3-28].

All patients were provided with individualized dietary guidance from a specialist dietitian, with a focus on calorie and fat restriction. For weight loss, a daily calorie deficit of 500 to 1000 kcal was calculated, alongside encouragement to increase physical activity. A low-fat diet, providing less than 20% to 25% of total energy from fat, was recommended to help improve insulin sensitivity and triglyceride levels. Patients were also advised to choose carbohydrate foods with a low glycemic index to aid glycemic control, and to include lean sources of protein with each meal and snack to promote satiety.

### Statistical analysis

Quantitative variables were summarized as median and interquartile range (IQR), as appropriate, and categorical variables as *n* (%). Normality was assessed with the Shapiro–Wilk test, and within-subject changes from baseline to the latest visit were analyzed using either a paired *t*-test or Wilcoxon matched-pairs signed-rank tests when appropriate. All descriptive and statistical analyses were performed using GraphPad Prism version 10.4.0 (GraphPad Software, Boston, MA).

## Results

In total, 40 patients were included, with females comprising 87.5% of the cohort. The median age was 46.5 years (IQR 39 to 59) with a median follow-up of 9.5 months (IQR 7 to 11). The median tirzepatide dose was 7.5 mg (IQR 5 to 12.5), with 77.5% (*n* = 31) of patients having previously used at least one other GLP-RA medication in the past, including dulaglutide, liraglutide, rybelsus (semaglutide), or ozempic (semaglutide). All patients were known to have non-ketotic diabetes (100%), with 87.5% also manifesting hypertriglyceridemia. Baseline characteristics of all patients are summarized in Tables 1 and 2.

**Table 1:** Baseline characteristics of individuals with PLD treated with tirzepatide

n = 40	Median [IQR]
Age	47.0 [39-59]
Follow-up duration (months)	9 [7-11]
Tirzepatide dose (mg)	7.5 [5-12.5]
Diabetes duration (years)	17 [10-23]
Sex	Female: 35 (87.5%)
	Male: 5 (12.5%)
FPLD type	FPLD2 = 4 (10%)
	FPLD3 = 7 (17.5%)
	FPLD7 = 1 (2.5%)
	APL = 1 (2.5%)
	FPLD1 like = 23 (65%)
	Unclassified*^a^* = 1 (2.5%)

Values are median and interquartile range (IQR).

Abbreviations: APL, acquired partial lipodystrophy; FPLD, familial partial lipodystrophy; FPLD1, like relates to participants with clinically detected limb lipodystrophy and preserved or increased central adiposity.

^*a*^Patient declined genetic testing.

**Table 2:** Baseline comorbidities among 40 individuals with PLD treated with tirzepatide

Comorbidity	n	%
**Metabolic/endocrine**		
Diabetes	40	100
Hypertriglyceridemia	35	87.5
Hypertension	30	75
PCOS	14	35*^a^*
**Cardiovascular/renal**		
Cardiac disease	5	12.5
PVD/Stroke	3	7.5
Renal disease	6	15
**Hepatic/pancreatic**		
MASLD	33	82.5
Cirrhosis	3	7.5
Pancreatitis	3	7.5
**Others**		
Retinopathy	24*^b^*	63.2
Obstructive sleep apnea (OSA)	8	20

Values are the total number of individuals (*n*) and the percentage of the total study group (%).

^*a*^14 out of 35 women.

^*b*^24 out of 38 individuals where diabetes retinal screening data were available.

### Treatment response following tirzepatide

**Figure 1:**
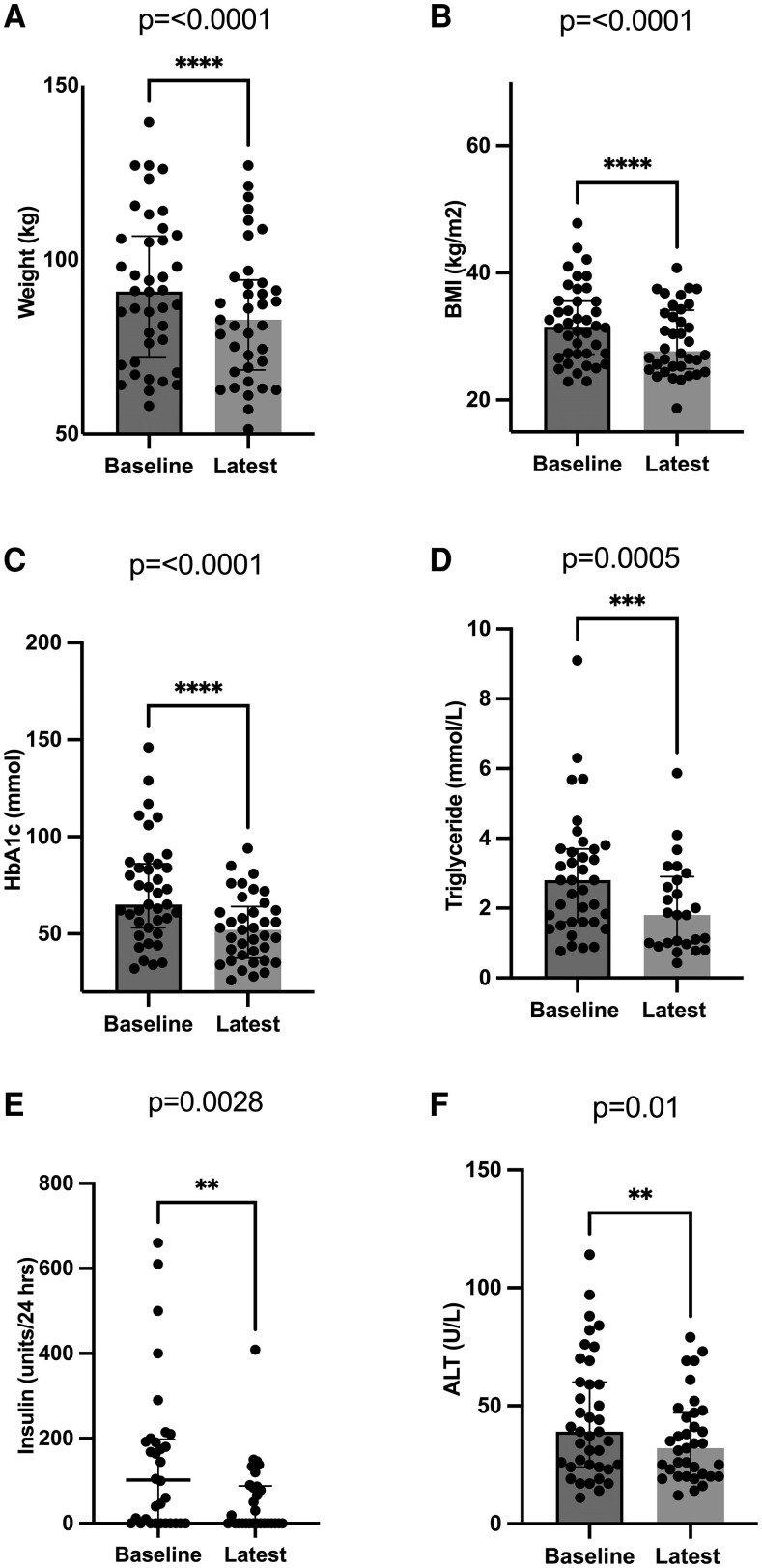
Changes in key variables in response to tirzepatide. (A) Change in weight (kg). (B) Change in BMI (kg/m2). (C) Change in HbA1c (mmol/L). (D) Change in serum triglyceride (mmol/L). (E) Change in total daily insulin use (units/24 hours). (F) Change in ALT (U/L). Bars represent median ± IQR, with each dot representing an individual participant. Paired t-test used for weight, BMI, and HbA1c. The Wilcoxon signed rank test was used for triglyceride, ALT, and insulin use per 24 hours.

Following treatment with tirzepatide, statistically significant reductions in weight and BMI were seen (Fig. 1A and 1B). Median body weight reduced from 91.0 kg (IQR 75.0 to 106.0) to 82.8 kg (IQR 69.0 to 93.4; *P* < .0001) and BMI fell from 31.5 kg/m^2^ (IQR 27.3 to 35.5) to 27.7 kg/m^2^ (IQR 24.7 to 33.8; *P* < .0001), respectively, equaling a total body weight loss percentage (TBWL %) of 7.9% (IQR −14.2 to −2.1, range −30.9% to +4.7%) at median follow-up of 9.5 months. This was associated with substantial reductions in HbA1c, which fell from 65 mmol/mol (IQR 54.5 to 85) to 52 mmol/mol (IQR 39 to 62; *P* < .0001) and serum triglycerides from 2.80 mmol/L (IQR 1.6 to 3.7) to 1.83 mmol/L (IQR 1.0 to 2.9; *P* < .0005) (Fig. 1C and 1D). Of the 40 patients in the study, 33 (82.5%) had an HbA1c above the diagnostic threshold for type 2 diabetes (> 48 mmol/mol) at baseline. From those 33 individuals, 12 (36.4%) were noted to have HbA1c fall to below the “diabetic cutoff” of 48 mmol/mol, and this occurred despite median daily insulin requirements being reduced by >50% (177.5 IU/24 hours (IQR 101 to 213.8) to 58.0 IU/24 hours (IQR 0 to 112.5; *P* = .0028)(Fig. 1E). Notably, 18% of patients established on insulin therapy were able to discontinue insulin completely following treatment with tirzepatide. The use of both oral hypoglycemic and lipid-lowering therapies was unchanged (*P* = .22 and *P*=>.9, respectively). 82.5% of our cohort had a diagnosis of metabolic dysfunction-associated steatotic liver disease (MASLD). Following treatment with tirzepatide, there was a statistically significant reduction in serum alanine aminotransferase (ALT) from 39 U/L (IQR 24.5 to 59.5) to 32 U/L (IQR 20.5 to 46.0; *P* = .01), suggestive of improved hepatic steatosis and potentially steatohepatitis (Fig. 1F). In contrast, no significant reduction in aspartate aminotransferase (AST) was observed, with a median baseline of 31 U/L (IQR 23.0 to 49.0) to 27 U/L (IQR 17.0 to 27.0; *P* = .21) at the latest follow-up, which is likely reflecting limited power as paired AST values were only available for 25% of the cohort. Consistent with this, although median FIB-4 scores decreased, the change did not reach statistical significance (*P* = .44) due to the restricted availability of AST measurements. A full description of anthropometric, metabolic, and medication changes can be seen in Table 3.

**Table 3:** Anthropometric, metabolic, and medication changes of individuals with PLD treated with tirzepatide

Variable	Baseline (median [IQR])	Latest (median [IQR])	Median percentage change (%)	Median difference [IQR]	Range	*P*-value
Weight (kg)	91.0 [75.0 to 106.0]	82.8 [69.0 to 93.4]	−9.0	−5.7 [−12.8 to −1.6]	−32.4 to +3.2	<.0001*
BMI (kg/m^2^)	31.5 [27.3 to 35.5]	27.7 [24.7 to 33.8]	−12.1	−1.7 [−5.8 to -0.7]	−12.9 to +1.1	<.0001*
TBWL%			−7.9 [−14.2 to −2.1]		−30.9 to +4.7	
*^a^* Triglycerides (mmol/L)	2.80 [1.6 to 3.7]	1.83 [1.0 to 2.9]	−34.6	−0.55 [−1.2 to −0.16]	−31.2 to +1.2	.0005*
HbA1c (mmol)	65 [54.5 to 85]	52 [39 to 62]	−20.0	−13 [−27 to −8]	−72 to +10	<.0001*
ALT (U/L)	39 [24.5 to 59.5]	32 [20.5 to 46.0]	−17.9	−5.0 [−16.5 to +2.0]	−53 to +20	*.01
AST (U/L)	31 [23.0 to 49.0]	27 [17.0 to 27.0]	−12.9	−4.0 [−13.0 to −0.5]	−25 to +8	.*21*
Creatinine (µmol/L)	56 [49.5 to 71]	58 [50 to 71.8]	3.6	−0.5 [−8 to +8.75]	−30 to +23	.*95*
FIB-4	1.11 [0.8 to 1.2]	0.83 [0.2 to 0.9]	−25.2		−0.51 to +0.26	.*44*
**Changes in medication**						
*^b^* Insulin use (I/U) per 24 hours	177.5 [101.3 to 213.8]	58.0 [0 to 112.5]	−67.3	−68.5 [−189.5 to −24.0]	−660 to +119	.0028*
Number of oral hypoglycemic agents	1.0 [1.0 to 2.0]	1.0 [1.0 to 2.0]	0	0.0 [0.0 to 0.0]	0 to 4	.*22*
Number of lipid-lowering agents	1.0 [0.0 to 1.0]	1.0 [0.0 to 1.0]	0	0.0 [0.0 to 0.0]	0 to 2	*>.9*

Values are shown as median [IQR]. Significance set to *P* < .05 (*). Bold italic values indicate statistically significant differences. Paired *t*-test used for weight, BMI, HbA1c, AST, and FIB-4. The Wilcoxon signed rank test was used for triglyceride, ALT, creatinine, insulin use per 24 hours, number of oral hypoglycemic agents, and number of lipid-lowering agents.

Abbreviation: TBWL, total body weight loss.

^*a*^One individual had severe hypertriglyceridemia at baseline (39.6 mmol/L), which improved by the latest follow-up to 8.4 mmol/L. Sensitivity analysis excluding this value did not meaningfully alter the summary statistics; therefore, the value was retained in the analyses.

^*b*^Values shown for participants receiving insulin at either time point.

Baseline data regarding retinopathy were available for 38 out of the 40 participants in our cohort. However, complete longitudinal data were available for 24 (60%) participants. Median baseline HbA1c at time of eye screening before treatment with tirzepatide was 66.5 mmol/mol (IQR 52 to 73), compared with 53 mmol/mol (IQR 42 to 62.5) at the time of post-treatment screening. The median interval from tirzepatide initiation to repeat eye screening was 11.5 months (IQR 6.5 to 14.25). Worsening diabetic eye disease was observed in 4 individuals (16.7%), all of whom developed new maculopathy in one eye. A further 3 individuals (12.5%) were noted to have developed new background retinopathy in either one or both eyes, but no participants developed new proliferative retinopathy. Improvements were observed in 4 individuals (16.7%), including resolution of maculopathy in one or both eyes in 3 participants and regression of background retinopathy in one participant. In the remaining 13 participants, no changes at screening following treatment with tirzepatide were observed, including 4 who had proliferative retinopathy at baseline.

In our cohort of PLD patients, there were 7 individuals with FPLD3, 4 with FPLD2, 1 with FPLD7, and 1 with acquired PLD (APL). All the others had central adiposity with limb lipodystrophy, ie, a phenotype similar to that widely known as FPLD1 or Kobberling type PLD (1, 22). In the participant with APL, tirzepatide was stopped after 4 months of treatment due to significant nausea and constipation. However, it was noted that this individual was started on 5 mg rather than 2.5 mg due to supply issues, which may be a factor in why the patient was unable to tolerate tirzepatide. Regardless, the patient was able to achieve a substantial reduction in HbA1c from 71 mmol/mol to 58 mmol/mol in 4 months despite reducing from 4 to 3 oral antidiabetic agents. Unfortunately, no up-to-date weight or serum triglyceride levels were available since stopping tirzepatide.

Tirzepatide was reasonably well tolerated, with a total of 6 participants (15%) having to stop treatment. In those where treatment was stopped (*n* = 6), 5 were due to gastrointestinal (GI) intolerance and one developed pancreatitis. Three participants required dose reduction, with one individual due to hypoglycemia and the remaining 2 due to new GI side effects after dose escalation. The individual who developed hypoglycemia had been receiving metformin, pioglitazone, and dapagliflozin. All 3 agents were discontinued following this episode of hypoglycemia on tirzepatide. Adverse event data were only available for 70% of patients (28 out of 40). Among those with available data, nausea and diarrhea were the most commonly reported side effects (46.4% and 42.9%, respectively). 32.1% described sulfur-smelling eructation, with 21.4% noticing constipation and 28.6% having had abdominal cramps. Most patients described these symptoms as having improved over time. Two participants (7.1%) described palpitations, but these settled without any medical intervention. One patient (2.5%) with FPLD3 developed pancreatitis in our cohort. Her serum triglycerides were 0.86 mmol/L on admission, and gallstones were excluded radiologically. This patient had described symptoms suggestive of possible previous undiagnosed pancreatitis on 2 occasions in the past. She reported severe abdominal pain radiating to her back one day after her third tirzepatide dose (2.5 mg), and pancreatitis was confirmed biochemically (serum amylase 179 U/L and serum lipase 360 U/L). Cross-sectional imaging revealed dilated common bile and pancreatic ducts, and subsequent endoscopic ultrasound was suggestive of chronic pancreatitis. Tirzepatide was stopped on admission, and the patient made a full recovery with no complications of pancreatitis detected on serial imaging. Although GI side effects seemed less frequent at higher doses for those who reported such effects, this is plausibly explained by selection bias, with only those who better tolerated tirzepatide advancing to the higher doses.

## Discussion

We retrospectively assessed the impact of tirzepatide in 40 individuals with PLD treated in a specialist clinic for patients with lipodystrophy. Substantial weight loss was observed at a median follow-up of 9.5 months. This weight loss was associated with very significant reductions in both HbA1c and serum triglycerides. These improvements occurred despite substantial (>50%) reductions in insulin use. Overall, tirzepatide was tolerated by most patients, but one participant did develop pancreatitis. Although this represents 2.5% of our cohort, making the proportion appear higher than the estimated background annual incidence in the general population (0.28%), interpretation is limited by our small sample size. Notably, this individual provided a clinical history suggestive of previous episodes of pancreatitis and was found to have radiological and endoscopic features consistent with chronic pancreatitis. Although almost half the participants in our cohort described gastrointestinal side effects, this is consistent with previously published data. For example, in the SURPASS-1 trial, evaluating the efficacy of tirzepatide in patients with T2DM, >1 treatment-emergent adverse event was observed in 66% of participants (24). Similarly, in trials of tirzepatide treatment for obesity, >1 adverse event was reported in 81% at 5 and 10 mg with 78.9% at 15 mg, respectively (25). Early worsening of diabetic retinopathy (EWDR) is a well-recognized phenomenon that occurs following rapid improvement of glycemia (26). It is thought to be transient and has been reported to occur in 10% to 20% of patients within 3 to 6 months (26). Moreover, Buckley et al (2025) recently reported real-world data in diabetic individuals treated with tirzepatide. Tirzepatide was found to result in significantly increased odds of incident proliferative diabetic retinopathy (PDR), with new onset PDR occurring in 1.1% of those exposed to tirzepatide compared to 0.5% in those unexposed (27). In our cohort, no participants developed new PDR, but 4 individuals (16.7%) did develop new maculopathy in one eye. Reassuringly, resolution of maculopathy was observed in 3 participants (12.5%). Amongst those who had proliferative retinopathy at baseline, no progression was observed. In the DCCT trial, which was a multicenter, randomized clinical trial comparing intensive vs conventional (1 to 2 daily insulin injections) diabetes treatment in insulin-dependent diabetic patients who had no to moderate nonproliferative retinopathy, early worsening was observed in 13.1% of patients assigned to intensive diabetes treatment (28). Given our small sample size, it is important to highlight that our findings should be interpreted with caution. Nonetheless, our findings do underscore the importance of retinal screening when treating with tirzepatide.

Metreleptin, which is a human leptin analogue, is the only drug to have received European regulatory approval for the treatment of lipodystrophy. Both the short and long-term benefits of metreleptin therapy in those with GLD have been well established (8, 29), but in those with PLD, the effects appear to be more modest, raising questions in terms of clinical benefit and cost-effectiveness (9, 30). In fact, in the study by Mosbah et al (2022), leptin therapy was not associated with sustained improvements in either HbA1c or triglycerides in patients with PLD (30).

Older generation GLP1-RAs, such as liraglutide (Victoza, Saxenda), have been described to improve glycemic control and reduce weight in patients with nonketotic diabetes and lipodystrophy (14, 18). Newer generation once-weekly GLP1-RAs, including dulaglutide (Trulicity) and semaglutide (Rybelsus, Ozempic, Wegovy), have built upon the efficacy of earlier agents by offering improved glycemic control and greater weight reduction (31, 32). Bhat et al (2023) described the use of dulaglutide in 2 individuals with PLD (one with FPLD1 and a second with FPLD6) (16). In the patient with FPLD1, dulaglutide treatment resulted in a 15.4% reduction in body weight and a 33.7% decrease in HbA1c after 4 months (16). Following the subsequent addition of atorvastatin, serum triglyceride concentrations declined by 68.7%.

Two retrospective studies by Lamothe et al (2024) and Foss-Freitas et al (2024) demonstrated that GLP1-RA resulted in significant improvements in body weight, HbA1c, and serum triglycerides in individuals with PLD (14, 15).

Tirzepatide has demonstrated superior efficacy compared with semaglutide in people living with obesity (19). Meral et al (2025) recently reported the use of tirzepatide in 17 patients with lipodystrophy, including 14 with PLD (7 with FPLD1, 6 with FPLD2, and 1 with FPLD3), after a median of 8.7 months of follow-up (33). Consistent with our findings, statistically significant reductions were observed in BMI (median, −1.7 kg/m^2^, *P* = .008), HbA1c (median, −1.1%, *P* < .001), serum triglycerides (median −65 mg/dL [−0.73 mmol/L], *P* = .003) and daily insulin use (median −109 units/day, *P* = .002) (33). However, the number of patients included in this study was limited to 14 individuals with PLD (33).

In our study, the median tirzepatide dose was 7.5 mg with a follow-up duration of 9.5 months. The mean total body weight reduction in our cohort was 7.9% at 9.5 months, which is comparable to the weight loss observed with the 5 mg dose in the SURPASS trials. These studies evaluated the efficacy of tirzepatide in patients with T2DM (SURPASS-1) and compared tirzepatide to semaglutide or insulin glargine in patients with T2DM (SURPASS-2 and 4) (24, 34, 35). However, our follow-up duration was shorter, and our cohort was relatively small. Mean HbA1c decreased by 19.1 mmol/mol (equivalent to a 1.75% point absolute reduction, from 71.9 to 52.8 mmol/mol; 26.6% relative reduction). In comparison, the SURPASS-1 trial reported absolute reductions in HbA1c of 1.87% with 5 mg and 1.89% at 10 mg, respectively (24). The mean baseline HbA1c in our cohort (71.9 mmol/mol) was higher than that in the SURPASS-1 trial (63.60 mmol/mol). Mean serum triglyceride level in our cohort, reduced by 1.61 mmol/L (41.5% relative reduction from 3.87 to 2.26 mmol/L) compared to 18.5% and 18.2% observed in the SURPASS-1 trial at 5 and 10 mg doses, respectively (24). This more pronounced reduction in serum triglyceride may plausibly be a difference in pathophysiology in those with PLD and potentially in background therapy in this patient population.

In our cohort, 27.5% (*n* = 11) were classed as overweight (BMI >25 kg/m^2^) and 62.5% (*n* = 25) classed as obese (BMI >30 kg/m^2^) in addition to their partial lipodystrophy, raising 2 important questions that remain unanswered. First, is tirzepatide effective in improving metabolic disease in PLD patients who are not overweight? Second, in those with PLD who do not have diabetes, does treatment with tirzepatide also lead to metabolic benefit?

Our study had several limitations. First, the relatively small sample size limits statistical power, which was evident for outcomes such as AST and FIB-4, where paired measurements were not available for all patients. In addition, adverse event data were only available from 28 out of 40 participants (70%). In view of this, some clinically important outcomes may not have reached statistical significance. Moreover, because not all patients may have been asked about side effects, there is a potential for reporting bias. Second, as this was a retrospective study, reliance was based on previously collected clinical data, which may introduce the potential for information bias, as data may have been incomplete. In addition, as there was no control group, we cannot exclude other factors, such as lifestyle changes, contributing to the findings. Lastly, the median follow-up for our study was relatively short; therefore, we were unable to evaluate the long-term effects of tirzepatide in this study. Despite these limitations, the improvements seen in several metabolic markers strengthen the validity of our findings and provide real-world data on the therapeutic impact of a relatively widely available drug in people with a rare disease. Although our study population consisted mostly of females with PLD, the observed metabolic improvements with tirzepatide are consistent with those reported in populations with obesity and type 2 diabetes. Therefore, the findings may potentially also extend to other forms of lipodystrophy, including generalized lipodystrophy. Our study included relatively few patients with FPLD2 and FPLD3, so further work will be required to clarify the impact of tirzepatide in patients with these subtypes of PLD.

## Conclusion

Our study suggests that in people with PLD, treatment with tirzepatide may provide substantial metabolic benefits for the treatment of diabetes and hypertriglyceridemia. Moreover, tirzepatide appears to be relatively well-tolerated. We would recommend further prospective studies to support our findings.

## Data Availability

Restrictions apply to the availability of some or all data generated or analyzed during this study to preserve patient confidentiality or because they were used under license. The corresponding author will on request detail the restrictions and any conditions under which access to some data may be provided.
